# A Biochemical and Pharmacological Characterization of Phospholipase A_2_ and Metalloproteinase Fractions from Eastern Russell’s Viper (*Daboia siamensis*) Venom: Two Major Components Associated with Acute Kidney Injury

**DOI:** 10.3390/toxins13080521

**Published:** 2021-07-26

**Authors:** Janeyuth Chaisakul, Orawan Khow, Kulachet Wiwatwarayos, Muhamad Rusdi Ahmad Rusmili, Watcharamon Prasert, Iekhsan Othman, Syafiq Asnawi Zainal Abidin, Mongkon Charoenpitakchai, Wayne C. Hodgson, Lawan Chanhome, Narongsak Chaiyabutr

**Affiliations:** 1 Department of Pharmacology, Phramongkutklao College of Medicine, Bangkok 10400, Thailand; vascharamon4321@gmail.com; 2 Queen Saovabha Memorial Institute, Thai Red Cross Society, Bangkok 10330, Thailand; okhow_2000@yahoo.com (O.K.); lchanhome@yahoo.com (L.C.); Narongsak.C@chula.ac.th (N.C.); 3 Institute of Pathology, Ministry of Public Health, Bangkok 10400, Thailand; kulachet4@gmail.com; 4 Kulliyyah of Pharmacy, International Islamic University Malaysia, Bandar Indera Mahkota, Kuantan 25200, Malaysia; rusdirusmili@iium.edu.my; 5 Jeffrey Cheah School of Medicine and Health Sciences, Monash University Malaysia, Bandar Sunway 46150, Malaysia; iekhsan.othman@monash.edu (I.O.); syafiqnawi@gmail.com (S.A.Z.A.); 6 Department of Pathology, Phramongkutklao College of Medicine, Bangkok 10400, Thailand; dr.cmongkon@gmail.com; 7 Monash Venom Group, Department of Pharmacology, Biomedical Discovery Institute, Monash University, Clayton, VIC 3800, Australia; wayne.hodgson@monash.edu

**Keywords:** phospholipase A_2_, venom, nephrotoxicity, Russell’s viper, myotoxicity, kidney

## Abstract

Acute kidney injury (AKI) following Eastern Russell’s viper (*Daboia siamensis*) envenoming is a significant symptom in systemically envenomed victims. A number of venom components have been identified as causing the nephrotoxicity which leads to AKI. However, the precise mechanism of nephrotoxicity caused by these toxins is still unclear. In the present study, we purified two proteins from *D. siamensis* venom, namely RvPLA_2_ and RvMP. Protein identification using LCMS/MS confirmed the identity of RvPLA_2_ to be snake venom phospholipase A_2_ (SVPLA_2_) from Thai *D. siamensis* venom, whereas RvMP exhibited the presence of a factor X activator with two subunits. In vitro and in vivo pharmacological studies demonstrated myotoxicity and histopathological changes of kidney, heart, and spleen. RvPLA_2_ (3–10 µg/mL) caused inhibition of direct twitches of the chick biventer cervicis muscle preparation. After administration of RvPLA_2_ or RvMP (300 µg/kg, i.p.) for 24 h, diffuse glomerular congestion and tubular injury with minor loss of brush border were detected in envenomed mice. RvPLA_2_ and RvMP (300 µg/kg; i.p.) also induced congestion and tissue inflammation of heart muscle as well as diffuse congestion of mouse spleen. This study showed the significant roles of PLA_2_ and SVMP in snake bite envenoming caused by Thai *D. siamensis* and their similarities with observed clinical manifestations in envenomed victims. This study also indicated that there is a need to reevaluate the current treatment strategies for Thai *D. siamensis* envenoming, given the potential for irreversible nephrotoxicity.

## 1. Introduction

*Daboia* spp. (Russell’s vipers) are a widely distributed snake genera in Asia and responsible for a high incidence of morbidity and mortality [1]. In 2016, the World Health Organization (WHO) classified Russell’s vipers as category 1 medically important venomous snakes in India, Nepal, Sri Lanka, Myanmar, Thailand and some Indonesian islands, i.e., Java, Komodo, Flores, and Lomblen [1]. Russell’s vipers are recognised as two distinct species, i.e., *Daboia russelii* (the Western Russell’s viper) and *Daboia siamensis* (the Eastern Russell’s viper). *D. russelii* is found throughout India, Pakistan, Bangladesh, Sri Lanka, and the west Bay of Bengal, whilst *D. siamensis* has a wide distribution across Southeast Asia (i.e., Myanmar, Thailand, and some Indonesian Islands), and Southern China (i.e., Guangdong and Guangxi), including Taiwan [1,2,3].

Acute kidney injury (AKI) caused by disseminated intravascular coagulopathy is commonly seen following envenoming by *Daboia* spp. regardless of the geographical range of the envenoming snake [4,5,6,7,8]. In contrast, the presence of neurotoxicity and myotoxicity following *Daboia* spp. envenoming displays remarkable geographical variation. According to the available clinical data, hematotoxicity and nephrotoxicity are prominently observed following *D. siamensis* envenoming in Taiwan and Myanmar [9,10]. Whereas, envenoming by the Sri Lankan and South Indian population of *D. russelii* causes significant skeletal muscle paralysis and myoglobinuria [11,12,13]. These clinical outcomes have been attributed to the presence of neurotoxic and myotoxic snake venom phospholipase A_2_ (PLA_2_) in the venom of this *Daboia* spp. population.

A proteomic investigation demonstrated that more than 90% of *D. siamensis* venom proteins belong to the Kunitz-type serine protease inhibitors, PLA_2_, C-type lectin/lectin-like protein, serine protease, and metalloproteinase families [14]. PLA_2_ and snake venom metalloproteinase (SVMP) from the venoms of *Daboia* spp. have been shown to be predominately responsible for coagulopathy, nephrotoxicity, neurotoxicity, cytotoxicity, and myotoxicity [15,16,17,18]. Daborhagin-M and daborhagin-K are P-III hemorrhagic metalloproteinases isolated from Russell’s viper venoms from Myanmar and eastern India, respectively. These SVMPs both induce severe hemorrhagic symptoms in experimental animals and also hydrolyze the Aα-chain of fibrinogen, fibronectin, and type IV collagen in in vitro studies [19]. PLA_2_s from *Daboia* spp. cause multiple toxic activities. For example, a myotoxic PLA_2_ [20] and a pre-synaptic PLA_2_ neurotoxin, called U1-viperitoxin [10], both isolated from Sri Lankan Russell’s viper (*D. russelii*) have been characterized in the chicken biventer preparation.

Recently, RvPLA_2_ and RvMP were isolated from *D. siamensis* venom and showed physiological function and histopathological changes in ex vivo perfused rabbit kidney model after incubation for 9 h [21]. However, potential contributing factors following administration of RvPLA_2_ and RvMP, for example, myotoxicity involvement, the effects of purified fractions in an in vivo model extending beyond 9 h, and their roles in the mechanism of AKI have not been fully investigated. The aim of the present study was to characterize RvPLA_2_ and RvMP from Thai *D. siamensis* venom using in vitro myotoxic and mice in vivo experiments. Protein characterization of RvPLA_2_ and RvMP was also undertaken to identify the amino acid sequences for both proteins.

## 2. Results

### 2.1. Identification of Purified Fractions

#### 2.1.1. Isolation and Purification of RvPLA_2_ and RvMP

**Figure 1:**
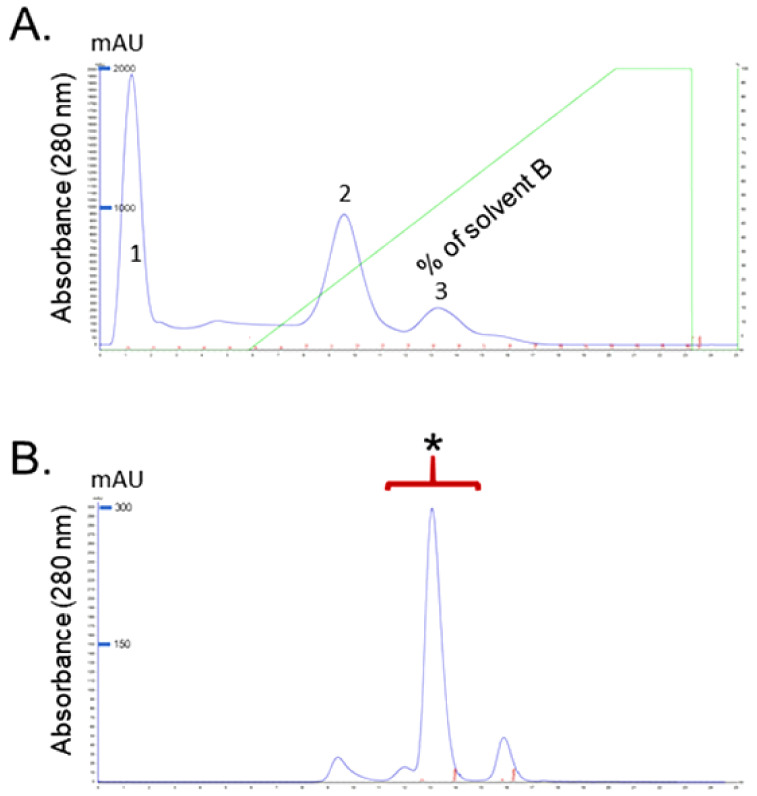
(**A**) A chromatogram of *Daboia siamensis* venom obtained via ion-exchange chromatography. Venom (25 mg) was applied to a Hitrap CMFF (1 mL) column equilibrated with 50 mM phosphate buffer pH 6.0, the elution was performed with a linear concentration gradient of NaCl from 0 to 1 M in the same buffer at a flow rate of 0.5 mL/min; (**B**) gel filtration of fraction II, the fraction II was subjected to a column of Superdex^TM^ 75 10/300 GL equilibrated with 10 mM PBS, pH 7.4. The flow rate was 0.5 mL/min.

Isolation and Purification of RvPLA_2_ and RvMP were completed as previously described [21]. In brief, for RvPLA_2_ isolation, *D. siamensis* venom (DSV) was fractionated using HiTrap CM FF ion-exchange column and the elution chromatogram yielded three major peaks (Figure 1A). Determination of PLA_2_ activity of the peaks showed that only fraction 2 contained PLA_2_ activity. This fraction was further purified using a Superdex^TM^ 75 10/300GL size exclusion column and the highest peak was chosen for further analysis (Figure 1B).

**Figure 2:**
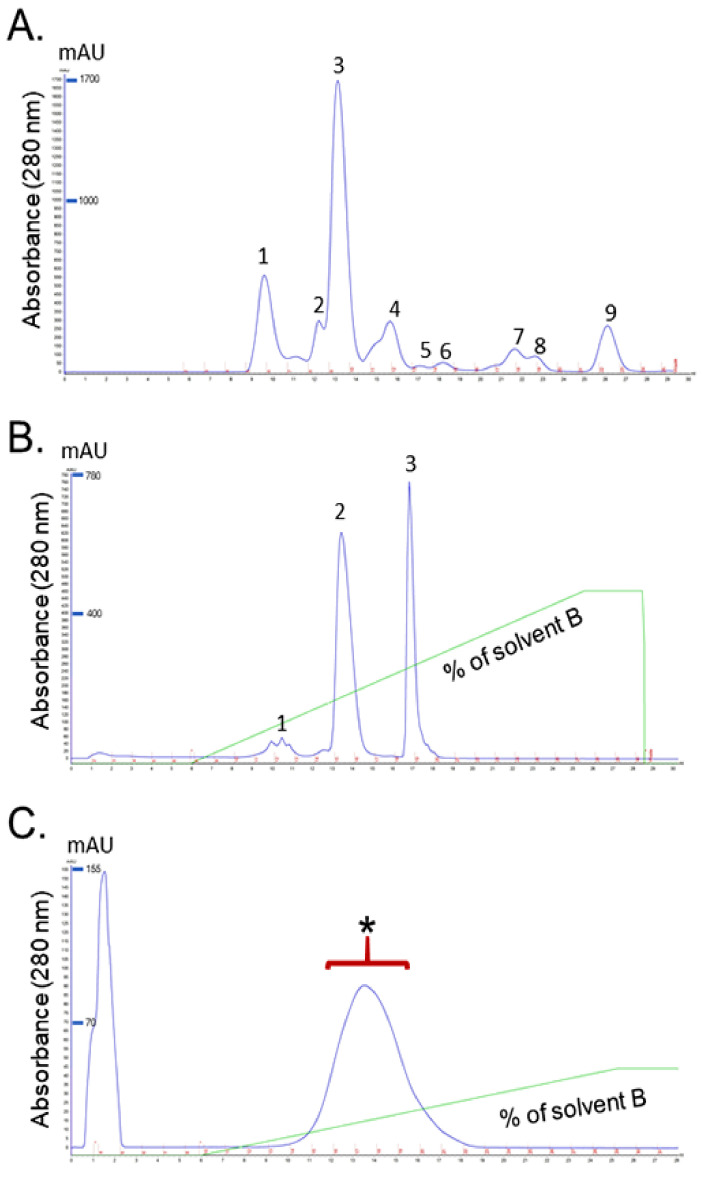
(**A**) Venom was applied on Superdex^TM^ 75 10/300GL column equilibrated with 0.1 M sodium acetate buffer, pH 6.7; (**B**) Peak 1 was then run on a Mono Q column (5/15 GL, GE Healthcare) pre-equilibrated with buffer A (50 mM Tris-HCl buffer, pH 8.0) and eluted with 60% linear gradient buffer B (1 M NaCl) as three fractions; (**C**) the active peak (i.e., 2nd of the three) was further purified by Resource S column which was pre-equilibrated with 10 mM sodium phosphate buffer, pH 6.7 and eluted with a linear gradient of 0–0.3 M NaCl.

For RvMP fractionation, *D. siamensis* venom was fractionated using a Superdex^TM^ 75 10/300GL size exclusion column and the elution chromatogram yielded nine peaks (Figure 2A). Fraction 1 was found to have the highest protease activity. Further purification of fraction 1, using a Mono Q column, yielded three main peaks and the highest protease activity was detected in Peak 2 (Figure 2B). Peak 2 was freeze-dried, and further fractionated using Resource S column. The second peak eluted from this step was chosen for further analysis (Figure 2C).

#### 2.1.2. Sodium Dodecyl Sulphate-Polyacrylamide Gel Electrophoresis (SDS-PAGE)

**Figure 3:**
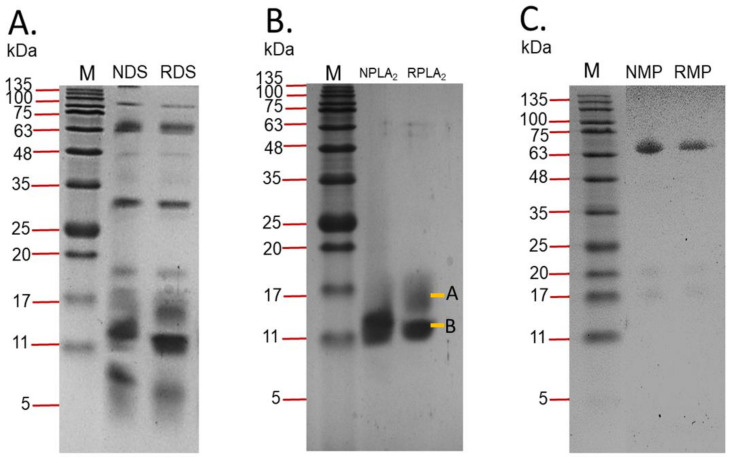
(**A**) SDS-PAGE of *D. siamensis* venom on a 15% separating gel with 5% stacking gel, venom was treated in reducing (RDS) or non-reducing buffer (NDS) prior to loading, electrophoresis, and stained with X-Press Blue Protein Stain; SDS-PAGE of (**B**) RvPLA_2_ and (**C**) RvMP on a 15% separating gel with 5% stacking gel (NPLA_2_/RvPLA_2_ in non-reducing buffer, RPLA_2_/RvPLA_2_ in reducing buffer (with two different bands, (**A**,**B**)), NMP/RvMP in non-reducing buffer, RMP/RvMP in reducing buffer, M is molecular weight marker).

*D. siamensis* venom, and the fractions of interest, were separated in a gel under non-reducing and reducing conditions (Figure 3). Thick and high intensity bands were observed in the MW range below 20 kDa in reduced and non-reduced venom. Several single protein bands of reduced and non-reduced venoms were also detected above the MW of 25 kDa (Figure 3A). The homogeneity and molecular weight of fractions were also assessed with SDS-PAGE. A thick protein band was detected in the lane loaded with non-reduced RvPLA_2_ with an estimated MW of 14 kDa. The two different bands, labeled as A and B, were detected in the lane loaded with reduced RVPLA_2_ and were estimated to have MW’s of 14 kDa and 12 kDa, respectively (Figure 3B). In lanes that were loaded with RvMP, the estimated molecular weights of RvMP under non-reducing and reducing conditions were 68 kDa and 65 kDa, respectively (Figure 3C).

#### 2.1.3. Phospholipase A_2_ Activity

The PLA_2_ activity of venom and fractions was determined using a secretory PLA_2_ colourmetric assay kit (Cayman Chemical, MI, USA). PLA_2_ activity for *D. siamensis* venom, RvPLA_2_, and RvMP were 2000 ± 245, 1620 ± 173, and 18 ± 7 µmol/min/mg (*n* = 3), respectively. The PLA_2_ activity of bee venom, used as a positive control, was 540 ± 22 µmol/min/mg (*n* = 3).

#### 2.1.4. Protein Identification for RvMP

**Figure 4:**
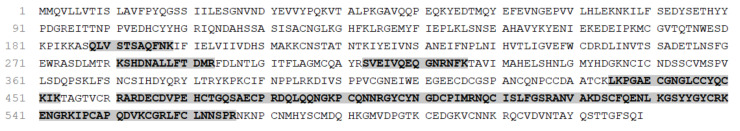
Sequence of coagulation factor X activating enzyme heavy chain (Q7LZ61). Amino acid sequences in grey are detected amino acids from LCMS/MS.

**Figure 5:**
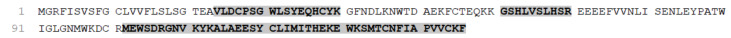
Sequence of snaclec coagulation factor X activating enzyme light chain (Q4PRD1). Amino acid sequences in grey are detected amino acids from LCMS/MS.

Protein identification and de novo sequencing showed RvMP to be identical to a factor X activator (Supplementary Data S1). Two proteins were detected in the sample: coagulation factor X activating enzyme heavy chain (UNIPROT accession number Q7LZ61, Figure 4) and snaclec coagulation factor X activating enzyme light chain (UNIPROT accession number Q4PRD1, Figure 5). The detected peptides showed 26% sequence similarity for the heavy chain and 36% for the light chain. Significant amino acid mutation was not detected from the de novo sequences.

#### 2.1.5. Protein Identification for RvPLA_2_

**Figure 6:**
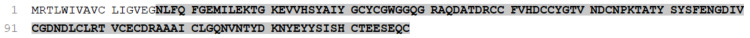
Amino acid sequence of acidic phospholipase A_2_ *Daboia siamensis* (P31100) in RvPLA_2_. Amino acid highlighted in grey were detected by LCMS/MS.

Protein identification by de novo sequencing from LCMS/MS analysis of the digested RvPLA_2_ peptides showed that they have 88% sequence similarity with acidic phospholipase A_2_ *Daboia siamensis* (UniPROT accession number P31100, Figure 6) (Supplementary Data S2). No amino acid mutation was detected.

### 2.2. Myotoxicity Studies

**Figure 7:**
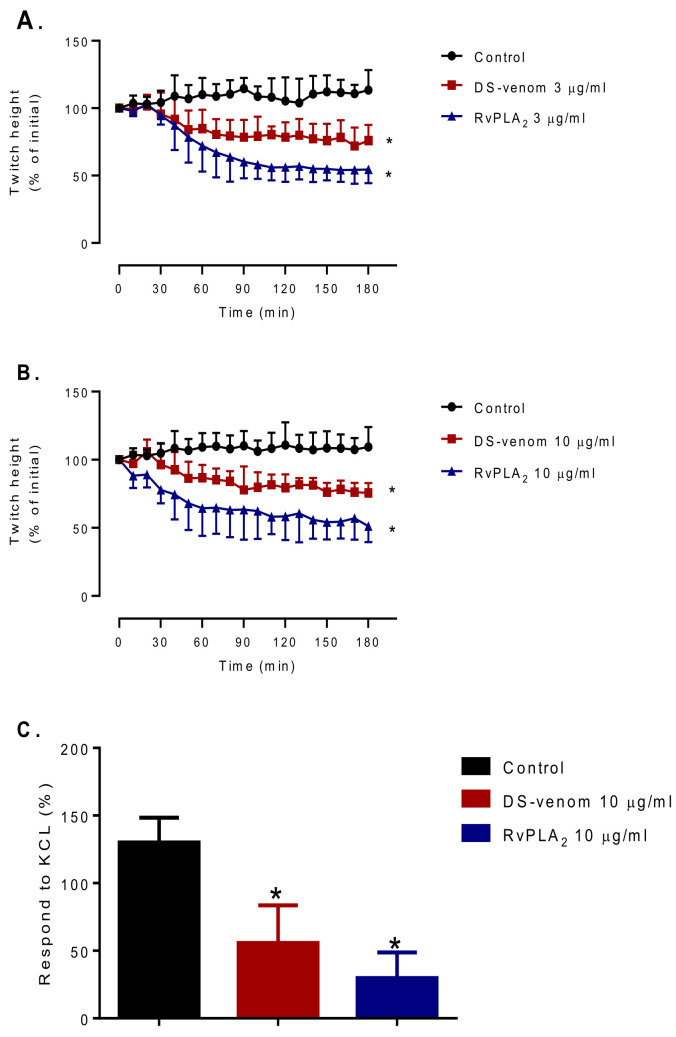
Effect of *D. siamensis* (DS) venom and RvPLA_2_ at 3 µg/mL (**A**) and 10 µg/mL (**B**) on direct twitches of the chick biventer cervicis nerve-muscle preparation including responses to KCL (40 mM for 30 s: (**C**). * Significantly different from vehicle control (*n* = 4, one-way ANOVA, *p* < 0.05).

*D. siamensis* venom (3–10 µg/mL, *n* = 4, one-way ANOVA, *p* < 0.05) and RvPLA_2_ (3–10 µg/mL, *n* = 4, one-way ANOVA, *p* < 0.05, Figure 7A,B) significantly reduced direct twitches in the chick biventer preparation as compared with vehicle. Neither venom (3–10 µg/mL, *n* = 4) nor RvPLA_2_ (3–10 µg/mL, *n* = 4, one-way ANOVA, *p* < 0.05) had a significant effect on baseline tension as compared with vehicle. Venom and RvPLA_2_ (3–10 µg/mL, *n* = 4, one-way ANOVA, *p* < 0.05) both significantly decreased contractile response to KCl as compared with the control (Figure 7C).

### 2.3. Histopathological Studies

**Figure 8:**
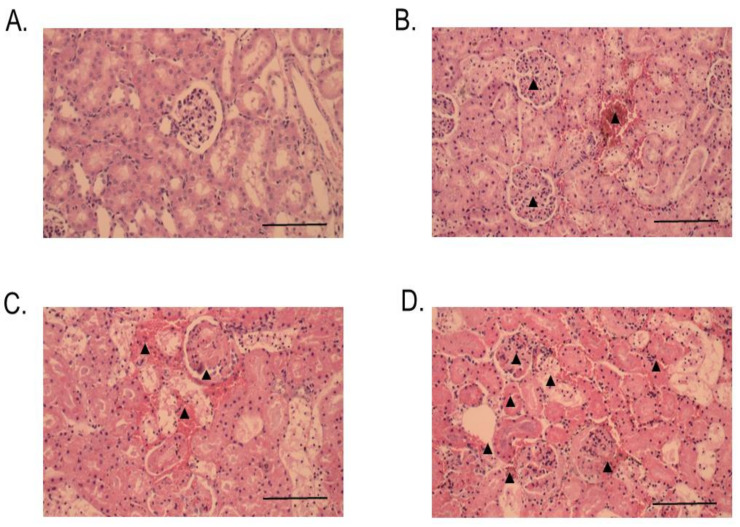
Histopathological changes of mice kidneys following 24-h intraperitoneal administration of: (**A**) Vehicle control; (**B**) Thai *D. siamensis* venom; (**C**) RvPLA_2_; (**D**) RvMP. H&E stain, 20× magnification. Scale bar = 50 µm. ▲ Indicates interstitial vessel congestion.

**Figure 9:**
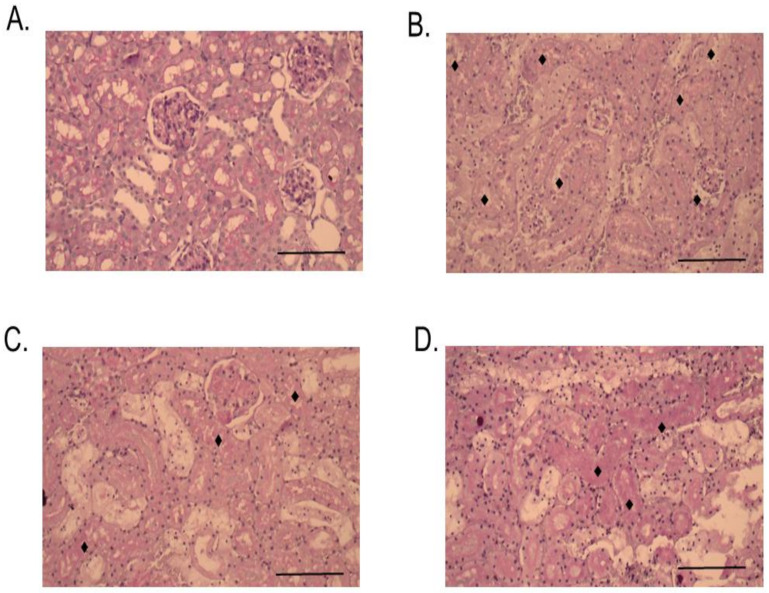
Histopathological changes of mice kidneys following 24-h intraperitoneal administration of: (**A**) Vehicle control; (**B**) Thai *D. siamensis* venom; (**C**) RvPLA_2_; (**D**) RvMP. PAS stain, 20× magnification. Scale bar = 50 µm. ♦ Indicates tubular injury.

Histopathological changes in mice caused by intraperitoneal (i.p.) administration of venom, RvPLA_2_, and RvMP were determined by light microscopic examination of kidney, heart, and spleen tissue samples. No significant histopathological change was observed in heart, kidney, and spleen tissues from animals that were administered with venom, RvPLA_2_, and RvMP at doses lower than 300 µg/kg (i.p.). Histopathological analysis of mice kidneys which were dissected at the 24 h time point following venom or RvPLA_2_ administration (300 µg/kg, i.p.) exhibited moderate (+2) morphological changes as compared with the negative control (Figure 8). Changes were defined by the presence of hyaline cast, dilatation of renal capillary, inflammatory foci, and glomeruli and/or congestion of interstitial vessels (Figure 8B,C). Severe congestion (+3) with hemorrhage was observed in glomeruli and interstitial vessels following the injection of RvMP (300 µg/kg, i.p.) after 24 h (Figure 8D). Administration of venom caused moderate tubular injury with partial loss of brush border with cell sloughing off and the presence of hyaline cast after 24 h (Figure 9B), whereas RvPLA_2_ and RvMP (300 µg/kg, i.p.) caused mild tubular injury (+1) with partial loss of brush border (Figure 9C,D).

**Figure 10:**
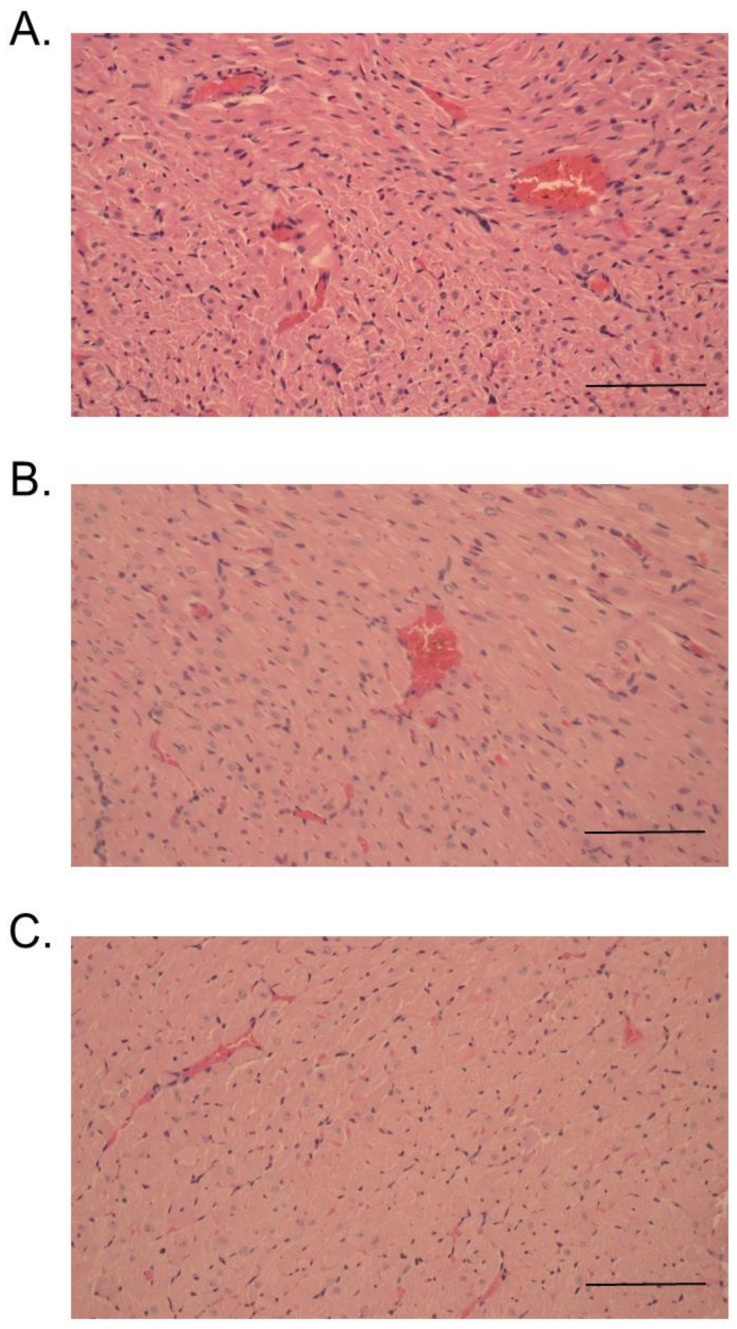
Histopathological changes of mice hearts following 24-h intraperitoneal administration of: (**A**) Thai *D. siamensis* venom; (**B**) RvPLA_2_; (**C**) RvMP. H&E stain, 20× magnification. Scale bar = 50 µm.

**Figure 11:**
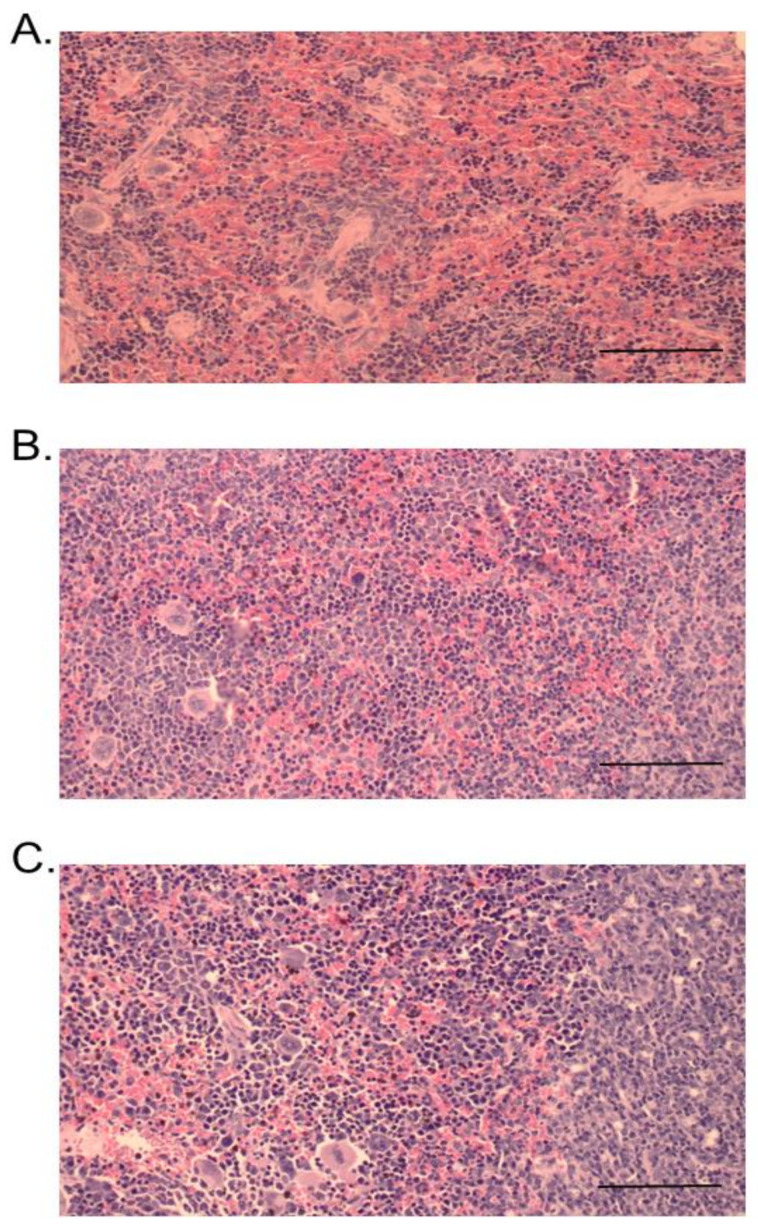
Histopathological changes of mice spleens following 24-h intraperitoneal administration of: (**A**) Thai *D. siamensis* venom; (**B**) RvPLA_2_; (**C**) RvMP. H&E stain, 20× magnification. Scale bar = 50 µm.

At 24 h, administration of venom or RvPLA_2_ (300 µg/kg, i.p.) caused a moderate degree (+2) of cardiac muscle necrosis and congestion (Figure 10A,B). A mild cardiac muscle necrosis was observed following administration of RvMP (Figure 10C). The spleen tissue samples for mice injected with venom and its fractions displayed hemorrhage and congestion 24 h after administration (Figure 11A–C).

## 3. Discussion

The present study demonstrated histopathological changes in tissues which are associated with the toxic activities of RvPLA_2_ and RvMP. We purified RvPLA_2_ and RvMP from Thai *D. siamensis* venom using ion-exchange and size-exclusion chromatography. The SDS-PAGE analysis detected two different protein bands of reducing RvPLA_2_, suggesting there might be some low molecular weight impurities in the sample. No band was observed at 25–35 kDa range in non-reduced RvPLA_2_ lane which exclude the possibility of the presence of a dimeric complex of PLA_2_. The identity of RvPLA_2_ was confirmed using LCMS/MS data which indicated that it was an acidic phospholipase A_2_ from *Daboia siamensis* with 88% sequence similarity. Previously, a purified anticancer PLA_2_ toxin, Drs-PLA_2_, was demonstrated to share 100% *N*-terminus sequence homology with a basic PLA_2_ from *D. r. siamensis* venom (viperotoxin). However, the pI for Drs-PLA_2_ was slightly more acidic, suggesting that viperotoxin was not Drs-PLA_2_ [22]. It was also shown that Drs-PLA_2_ had in vitro hemolytic, anticoagulant, and cytotoxicity activities. Although many Russell’s viper PLA_2_ toxins share a high degree of similarity in amino acid sequence, their enzymatic and other biological activities might be different due to slight differences in amino acid sequence. This could be due to the presence of post-translational modification of the proteins that was not able to be detected by the method used in the current study. This also indicates possible intra- and inter-species variation and a structure–function relationship of viperid venom PLA_2_ [23]. Snake venom metalloproteases (SVMPs) are significant components in most viper venoms, and cause hemostatic and cellular homeostasis disturbances. According to the presence and absence of various non proteinase domains, as observed via mRNA transcripts and proteins in the venom, SVMPs are categorized into PI, PII (a and b), and PIII (a and b) classes [24]. Functional variabilities of SVMPs were reported to indicate distinct mechanisms in the coagulation processes of human, avian, and rodents [25]. SVMPs from viper venoms displayed molecular weights between 20 and 70 kDa [25]. In the present study, the estimated molecular weight of RvMP from SDS-PAGE was 65 kDa when reduced, and 68 kDa when not reduced. RvMP is a PIII snake venom metalloproteinase based on its molecular weight, detected peptides, and matched protein. BnMPIIIc-SVMP, from *Bothrops neuwiedi* venom, also displayed a molecular weight of 67 kDa [25]. The matched protein, coagulation factor X activating enzyme heavy chain (Q7LZ61), was reported to exist as a heterotrimeric, with one heavy chain and two light chains linked by disulfide bonds [26]. However, based on the SDS-PAGE band profiles of reduced and non-reduced samples, RvMP does not possess other subunits. Although a snaclec coagulation factor X activating enzyme light chain (Q4PRD1) was also detected in the sample, it was not present in sufficient concentration to be detected by SDS-PAGE under non-reducing and reducing conditions but enough for the highly sensitive mass-spectrometry system. This indicates that it could be a co-eluted protein in the RvMP-containing fraction.

A recent study, using an ex vivo perfused rabbit kidney model, showed that RvPLA_2_ and RvMP (280 µg/mL) significantly increased perfusion pressure, urinary flow rate, glomerular filtration rate, osmolar clearance, and renal vascular resistance [21]. Histopathological determination of perfused rabbit kidney sections revealed crystal deposits in the glomerular capillary lumen, dilatation of proximal and distal convoluted tubules, as well as tubulonephrosis, following incubation with RvPLA_2_ or RvMP for 90 min. In the current study, we determined histopathological changes of mouse kidneys following intraperitoneal administration of venom or fractions (300 µg/kg) for 24 h. A moderate degree of histopathological lesions was detected as diffuse and/or focal glomeruli, and congestion of interstitial vessels and tubular injury. These are almost the same characteristics of kidney lesion as identified in previous studies using perfused rabbit kidneys [21] and rat in vivo experiments [27]. However, the degree of morphological changes observed in our present work could not be compared with previous work performed by our group due to the differences in species of experimental animal used and time of contact with venom or toxins. The degree of histopathologic change observed following snake envenoming is significantly associated with the time of contact with venom or toxin [28]. The effects of RvPLA_2_ and RvMP on physiological function and morphological changes were abolished by the prior addition of the platelet activating factor (PAF) inhibitor, WEB 2086 [21]. This suggests a role for PAF to modulate glomerular functions via PAF receptor within the renal glomeruli [29].

Acute kidney injury (AKI) appears to be an important clinical outcome associated with mortality, especially those bitten by *Daboia* species [10]. The pathogenesis of AKI after Russell’s viper envenoming is incompletely understood, but seems to involve microvascular fibrin deposition, direct nephrotoxicity, and cardiovascular impairments [30]. The mechanisms behind AKI following viper envenoming have been suggested to be associated with DIC or coagulopathy [31]. Here, we demonstrated that administration of *D. siamensis* venom, either RvPLA_2_ or RvMP, for 24 h to mice produced diffuse glomerular congestion, hyaline cast, and moderate tubular necrosis, suggesting Russell’s viper envenoming induces acute kidney injury [27]. Our histopathological data correlates with clinical observations in envenomed patients indicating that acute tubular necrosis as well as renal cortical necrosis become the predominant pathologies detected in kidney biopsy [32,33].

Myotoxicity with a significant increase in creatine kinase (CK) is a common clinical observation following envenoming by myotoxic snake species including sea snakes (e.g., *Hydrophiinae*), some elapids (e.g., *Pseudechis australis* and *Bungarus candidus*) [34,35] and some vipers (e.g., *Daboia russelii* and *Bothrops asper*) [9,36]. Systemic myotoxicity, which causes rhabdomyolysis, is induced by systemic myotoxins that cause widespread muscle injury resulting in elevation of CK, hyperkalaemia, and AKI with myoglobinuria. In the present study, we demonstrated that RvPLA_2_ from *D. siamensis* venom attenuated twitch height of directly stimulated chick biventer cervicis muscle and also decreased contractile response to KCl, both indicative of myotoxicity [37]. Previously, myotoxic PLA_2_ toxins, U1-viperitoxin-Dr1a and U1-viperitoxin-Dr-b, were purified from Sri Lankan *D. russelii* venom and exhibited mild myotoxic activity in anaesthetised experimentally envenomed animals and in vitro chick biventer muscle [20]. In fact, marked myotoxicity with skeletal muscle breakdown is likely to be absent following envenoming by *D. siamensis*, in agreement with a clinical observation in Sri Lanka indicating mild myotoxicity following *D. russelii* envenoming [20]. Indeed, the delayed onset of myotoxicity seen in envenomed patients, and its correlation with the pharmacokinetic profile of the venom and the rise in CK in envenomed patients, has been shown in a previous study [35].

Clinically, administration of antivenom is the only reliable treatment for snakebite-envenomed patients. The time interval from bite to antivenom administration is the strongest predictor of AKI [10]. Early administration of antivenom was associated with a shorter duration of coagulopathy and nephrotoxicity [10,27]. It has been shown that early administration of monovalent *D. siamensis* antivenom prevented a rise of BUN and creatinine in anaesthetized animals, and also prevented venom-induced morphological changes of kidneys [27]. Interesting, *D. siamensis* monovalent antivenom and Hemato polyvalent antivenom from the Thai Red Cross were able to interact with Sri Lankan *D. russelii* venom in immunobinding assays, suggesting cross neutralizing effect of these antivenoms for venoms from different species [38].

In the present study, in addition to AKI and nephrotoxicity, *D. siamensis* venom and its toxins (i.e., RvPLA_2_ and RvMP) also caused congestion of cardiac muscle and spleen. This indicates that systemic envenoming by *D. siamensis* venom also affects other organs. Clinically, myocardial infarction with ST elevation was reported in Sri Lanka in patients bitten by *D. russelii* [39] and *Hypnale hypnale* [40]. However, our knowledge of myocardial infarction and other systemic outcomes observed following viper envenoming is poorly understood. Indeed, the pathophysiology of the cardiovascular system following viper envenoming needs to be explored further.

In conclusion, RvPLA_2_ and RvMP from *D. siamensis* venom cause significant nephrotoxic outcomes. The myotoxic effect of RvPLA_2_ may contribute to the induction of myoglobinuria resulting in AKI. A study to determine the effectiveness of antivenom to prevent nephrotoxicity induced by these two fractions would be beneficial in order to develop treatment strategies for acute kidney injury in envenomed patients.

## 4. Materials and Methods

### 4.1. Snake Venoms

Venom was extracted from more than 20 captive specimens (both male and female) of Eastern Russell’s viper (*D. siamensis*) held at QSMI, The Thai Red Cross Society Bangkok, Thailand. Venoms were pooled, frozen, and then freeze-dried. Dried venoms were weighed and stored at −20 °C prior to use. The venom was reconstituted in phosphate-buffered saline (PBS) when needed, unless stated otherwise.

### 4.2. Protein Concentration

The protein concentration of venom and fractions was determined, as per manufacturer’s instructions, using a BCA Protein Assay Kit (Pierce Biotechnology, Rockford, IL, USA). In brief, venom (25 µL) was pipetted onto a 96-well plate in triplicate. Then, reagent buffer mix (200 µL) was added to each well. The plate was incubated at 37 °C for 30 min, and then analyzed at 562 nm using an ELISA plate reader spectrophotometer (Enspire^®^ multimode plate reader, Waltham, MA, USA). Protein concentration of the sample was determined from the bovine serum albumin standard curve.

### 4.3. Fractionation of Venom

Isolation and purification of snake venom phospholipase A_2_ (RvPLA_2_) and snake venom metalloproteinase (RvMP) were performed following previously described methods [21].

#### 4.3.1. Purification RvPLA_2_

*D. siamensis* venom was dissolved in buffer A (50 mM phosphate pH 6.0) and centrifugated at 10,000 rpm for 5 min. The supernatant was loaded onto a HiTrap CM FF column (GE Healthcare, Uppsala, Sweden). The column was equilibrated with 5 volumes of buffer A and elution was carried out with an increasing linear gradient of 0–1 M NaCl in buffer A at a flow rate of 0.5 mL/min for 15 column volumes. Absorbance of the elution was monitored at 280 nm and the eluted fractions were collected using an AKTA pure Fast Protein Liquid Chromatography system (GE Healthcare, Uppsala, Sweeden). Four peaks were obtained from the elution chromatogram and each peak was tested for PLA_2_ activity using the Holzer and Mackessy method [21,41]. Peaks with PLA_2_ activity were pooled and further fractionated using a Superdex^TM^ 75 10/300GL column (GE Healthcare, Uppsala, Sweden) mounted on a AKTA pure Fast Protein Liquid Chromatography system (GE Healthcare, Uppsala, Sweden). The fraction was eluted using 10 mM PBS, pH 7.4, at room temperature. The flow rate was set at 0.5 mL/min and 1 mL fraction was collected in each tube and elution was run for 50 min. The eluted proteins were detected by absorbance at 280 nm.

#### 4.3.2. Purification of RvMP

The venom supernatant was loaded onto a Superdex^TM^ 75 10/300GL column equilibrated with 0.1 M sodium acetate buffer pH 6.7. The elution was carried out using 0.1 M sodium acetate buffer pH 6.7 at a flow rate of 0.4 mL/min and fractions of 1 mL per tube were collected using an AKTA Pure FPLC system (GE Healthcare, Sweden) for 50 min. Each fraction was assayed for protease activity, as previously described [21,42]. The fractions with protease activity were collected, desalted, and concentrated by centrifugal ultrafiltration (Macrosep^®^ 10K, Pall Corp., Portsmouth, UK). Then, the pooled fraction was loaded onto a Mono Q column (5/15 GL, GE healthcare) washed with 5 column volumes of buffer A (50 mM Tris-HCl buffer, pH 8.0) and eluted with 0–60% linear gradient buffer B (1 M NaCl) for 20 column volumes. Three peaks were separated, the active peak was further purified by Resource S column which was equilibrated with 10 mM sodium phosphate buffer pH 6.7, and eluted with a linear gradient of 0–0.3 M NaCl. The eluted proteins were detected by absorbance at 280 nm.

### 4.4. Determination of PLA_2_ Activity

The PLA_2_ activity for venom and fraction was determined, according to the manufacturer’s instructions, using a secretory PLA_2_ colormetric assay kit (Cayman Chemical, Ann Harbor, MI, USA). In brief, the 1,2-dithio analog of diheptanoyl phosphatidylcholine was used as a substrate for venom PLA_2_ enzymes. Free thiols generated following the hydrolysis of the thio ester bond at the *sn*-2 position by PLA_2_ were detected using DTNB (5,5′-dithio-bis-(2-nitrobenzoic acid)). The change of absorbance was monitored at 405 nm and sampled every minute for 10 min period using a plate reader spectrophotometer (EnSpire^®^ Multimode Plate Reader, Perkin Elmer, MA, USA). The PLA_2_ activity was calculated as micromoles of phosphatidylcholine hydrolyzed per min per mg of enzyme. Determination of PLA_2_ activity was performed in triplicate for all samples including bee venom (positive control).

### 4.5. Sodium Dodecyl Sulphate-Polyacrylamide Gel Electrophoresis (SDS-PAGE)

Venom or fractions (10 μg) was mixed with reducing and non-reducing sample buffers before being loaded into 15% separating gel with 5% stacking gel. The gel was electrophoresed at 90 V for 30 min, and then 120–150 V for 1 h, using the method previously described [43]. TriColor Broad Protein Ladder (Biotechrabbit GmbH, Henigsdorf, Germany) was used as a protein molecular weight marker. Protein bands were visualized by staining the gel with X-Press Blue Protein Stain (Himedia, L.B.S. Marg, Mumbai, India), followed by de-staining using distilled water. Then, the gel was scanned using a Chemi Imager, Alliance Mini HD9 Auto (UVITEC, Cambridge, UK).

### 4.6. In-Solution Digestion of Protein

Protein digestion was performed using the method recommended by the mass spectrometry manufacturer (Agilent Technologies, Santa Clara, CA, USA). In brief, freeze dried samples were treated with ammonium bicarbonate (25 µL, 100 mM), trifluoroethanol (25 µL), and DTT (1 µL; 200 mM). This mixture was briefly vortexed, centrifuged, and then incubated at 60 °C for 1 h. Iodoacetamide (4 µL, 200 mM) was added into the tubes and left for 1 h in the dark. Then, DTT (1 µL) was added into the tubes and left for 1 h at room temperature. Prior to trypsin addition, the pH of the treated samples was adjusted to pH 7–9 using Milli-Q water and ammonium bicarbonate (100 mM). Then, the samples were incubated overnight at 37 °C. Formic acid was used to terminate the trypsin reaction at the end of the incubation. The samples were dried using a vacuum concentrator and stored at −20 °C prior to analysis. The sample was re-dissolved using 0.1% formic acid prior to loading into an ESI-LCMS/MS system.

### 4.7. Nanoflow Liquid Chromatography-Ionization Coupled with Mass Spectrometry/Mass Spectrometry (ESI-LCMS/MS)

The digested sample was loaded into an Agilent C18 300 Å Large Capacity Chip (Agilent Technologies, Santa Clara, CA, USA) mounted onto an Agilent 1200 HPLC-Chip/MS Interface, coupled with an Agilent 6550 iFunnel Q-ToF LC/MS (Agilent Technologies, Santa Clara, CA, USA). The flow rate was set at 4 µL/min and 0.5 µL/min for the capillary pump and the nano pump, respectively. The chip column was equilibrated with 0.1% formic acid in water and peptides were eluted with an increasing gradient of 90% ACN in 0.1% formic acid using the following gradient: 0–75% from 0 to 30 min and 75% for 4 min. The mass spectrometry was set at positive ion polarity mode, the capillary voltage was set at 2050 V, and the fragmentor voltage was set at 360 V. Gas temperature was 325 °C and drying gas flow was set at 5 L/min.

### 4.8. Main Venom Protein Identification

Samples were identified using a PEAK Studio software (version 7.0, Bioinformatics Solution, Waterloo, ON, Canada). The homology search was performed by comparing de novo sequence tags generated from the software using mass spectrometry data with UniProt Serpentes database from July 2017. In the software, carbamidomethylation was set as the fixed modification, trypsin as the digestion enzyme, and parent mass error tolerance and fragment mass error tolerance were set at 0.1 Da. The identity of the protein was accepted if they fulfilled the following criteria: the maximum number of missed cleavages and maximum variable pot-translational modification per peptide ≤3, false detection rate (FDR) <0.1%, the minimum value for protein -10logP is 30, and the minimum number of unique peptides is 2. In addition to these parameters, the identity of the protein was determined based on the source of organism of the matched protein, the highest -10logP value and the highest number of unique and matched peptides.

### 4.9. Animal Care and Ethics

All animal experiments were performed under protocols approved by the Subcommittee for Multidisciplinary Laboratory and Animal Usage of Phramongkutklao College of Medicine and the Institutional Review Board, Royal Thai Army (IRBRTA) Department, Bangkok, Thailand (Ethical Clearance code IRBRTA 456/2560, approval date 16 August 2017 and IRBRTA 222/2562, approval date 21 February 2019) in accordance with the UK Animal (Scientific Procedure) Act, 1986 and the National Institutes of Health guide for the care and use of Laboratory animals (NIH Publications No. 8023, revised 1978).

### 4.10. Chick Biventer Cervicis Nerve-Muscle Preparation for Myotoxicity Determination

Male chickens (*Gallus gallus domesticus*), aged between 4–10 days, were obtained from a local poultry hatchery (Bangkok, Thailand) and kept in a well-lit cage with access to food and drinking water ad libitum. Chickens were humanely killed by CO_2_ asphyxiation. The biventer cervicis nerve-muscles were both removed and mounted in 5 mL organ baths containing physiological salt solution of the following composition: 118.4 mM NaCl, 4.7 mM KCl, 1.2 mM MgSO_4_, 1.2 mM KH_2_PO_4_, 2.5 mM CaCl_2_, 25 mM NaHCO_3_, and 11.1 mM glucose. The solution was maintained at 34 °C and bubbled with carbogen (95% O_2_ and 5% CO_2_) under 1 g resting tension. Direct stimulation (rate 0.1 Hz and pulse duration 2 ms) was applied to tissues at supramaximal voltage (20–30 V) using a Grass SD9 stimulator with an electrode placed around the middle of the muscle. To ensure selective muscle stimulation, *d*-tubocurarine (10 µM) was added and left in the organ bath throughout the duration of experiment. Contractile responses to potassium chloride (KCl, 40 mM for 30 s) was obtained in the absence of electrical stimulation. In all experiments, venom or purified fractions (3–10 µg/mL) were left in contact with the preparation for up to 3 h. Twitch responses were measured using Grass force-displacement transducers (FT03) and recorded using a MacLab System. A significant contracture of skeletal muscle (i.e., a rise in baseline) and/or inhibition of direct twitches were considered as signs of myotoxicity.

### 4.11. Histopathological Effects of RvPLA2 and RvMP on Rodent Tissues

#### 4.11.1. Animal Treatments

Male Jcl:ICR mice, weighing between 20 and 30 g, were purchased from Nomura-Siam International Co. Ltd., Bangkok, Thailand. Animals were housed in stainless steel containers with access to food and drinking water ad libitum. RvPLA_2_ and RvMP dose 50, 150, and 300 µg/kg were chosen for preliminary test. *D. siamensis* venom, saline, RvPLA_2_, or RvMP was intraperitoneally (i.p.) administered to 3 rats per dose. All animals were humanely euthanized by cervical dislocation at 24 h after i.p. administration.

#### 4.11.2. Histopathological Studies

The kidneys, heart, and spleen were removed from euthanized animals and preserved in 10% formaldehyde before being embedded in paraffin. Embedded tissues were cut and stained with hematoxylin and eosine (H&E) and/or periodic acid Schiff (PAS). Tissue determination for morphological change was done under a light microscope (Olympus BH-2, Olympus Optical Co., Tokyo, Japan). Areas in the slide with morphological changes due to typical myotoxicity and nephrotoxicity were photographed using an Olympus C-35AD camera (Olympus Optical Co.).

### 4.12. Data Analysis and Statistics

Prism 6.0 software (GraphPad Software, La Jolla, CA, USA) was used for statistical analysis. Twitch height and contractile responses to KCl were expressed as a percentage of the corresponding value prior to the administration of venom or purified fractions. Multiple comparisons were done using a one-way analysis of variance (ANOVA) followed by a Bonferroni multiple comparison test. Values of *p* < 0.05 were accepted as significant. Data were expressed as mean ± SEM.

## Data Availability

Not applicable.

## Supplementary Materials

The following are available online at https://www.mdpi.com/article/10.3390/toxins13080521/s1, Data S1: Sequence of RvMP, Data S2: Sequence of RvPLA2.
