# Supplementary material for: A Biochemical and Pharmacological Characterization of Phospholipase A2 and Metalloproteinase Fractions from Eastern Russell’s Viper (Daboia siamensis) Venom: Two Major Components Associated with Acute Kidney Injury
Source: Toxins (Basel). 2021 Jul 26;13(8):521. doi: 10.3390/toxins13080521 (PMC8402394; doi:10.3390/toxins13080521)
Supplement: Supplementary file 1 [file toxins-13-00521-s001.zip › toxins-1300854-supplementary.pdf]

toxins

MDPI

Supplementary Materials: A Biochemical and Pharmacological Characterization of Phospholipase A_2 and Metalloproteinase Fractions from Eastern Russell's Viper (*Daboia siamensis*) Venom: Two Major Components Associated with Acute Kidney Injury

Janeyuth Chaisakul, Orawan Khow, Kulachet Wiwatwarayos, Muhamad Rusdi Ahmad Rusmili, Watcharamon Prasert, Iekhsan Othman, Syafiq Asnawi Zainal Abidin, Mongkon Charoenpitakchai, Wayne C. Hodgson, Lawan Chanhome and Narongsak Chaiyabutr

Supplementary Data S1: Sequence of RvMP

a. Protein identification for RvMP. Q7LZ61 was determined to be the identity of the heavy chain subunit identity and Q4PRD1 was determined as light chain subunit identity. The determination was made based on analysis of -10lgP value, number of unique peptides and matched origin species.

Protein Group	Protein ID	Accession	-10lgP	Coverage (%)	Coverage (%) SVMP	Area SVMP	#Peptides	#Unique	#Spec SVMP	PTM	Avg. Mass	Description
1	21019	Q7LZ61|VM3CX_DABSI	209.89	26	26	0	29	11	119	Y	69648	Coagulation factor X-activating enzyme heavy chain OS=Daboia siamensis PE=1 SV=2
2	21020	tr|K9JAW0|K9JAW0_DABRR	189.84	29	29	0	23	5	96	Y	69521	Factor X activator heavy chain OS=Daboia russelii PE=2 SV=1
3	21021	Q4PRD1|SLLC1_DABSI	149.91	36	36	0	9	9	36	Y	16871	Snaclec coagulation factor X-activating enzyme light chain 1 OS=Daboia siamensis GN=LC1 PE=1 SV=2
5	21022	tr|T1P647|T1P647_9SAUR	121.68	42	42	0	10	2	22	Y	18324	Factor X activator light chain 2 OS=Daboia russelii PE=2 SV=1
7	21036	tr|K9JDJ1|K9JDJ1_DABSI	108.36	52	52	0	10	2	15	Y	18479	Factor X activator light chain 2 OS=Daboia siamensis PE=2 SV=1
9	21042	tr|K9JCB2|K9JCB2_DABRR	104.50	18	18	0	3	2	8	Y	18273	Factor X activator light chain 2 OS=Daboia russelii PE=2 SV=1

i. Matched peptides for RvMP heavy chain subunit (Q7LZ61).

Supporting Peptides:

Peptide	Uniq	-10lgP	Mass	Length	ppm	m/z	z	RT	Scan	Area SVMP	#Feature	#Feature SVMP	Start	End	PTM	Found By
R.ANVAKDSC(+57.02)FQENLKGSYYGYC(+57.02)R.K	Y	62.51	2629.1689	22	-8.7	877.3893	3	7.42	1680	0	0	0	518	539	Carbamidomethylation	PEAKS DB
K.DSC(+57.02)FQENLKGSYYGYC(+57.02)R.K	N	59.31	2145.8884	17	-3.2	716.3011	3	7.83	1802	0	0	0	523	539	Carbamidomethylation	PEAKS DB
R.ANVAKDSC(+57.02)FQENLK.G	Y	55.11	1622.7722	14	2.1	812.3951	2	5.86	1184	0	0	0	518	531	Carbamidomethylation	PEAKS DB
R.ARDEC(+57.02)DVPEHC(+57.02)TGQSAEC(+57.02)PRDQLQQNGKPC(+57.02)QNNR.G	Y	51.44	4053.7283	34	6.3	811.7581	5	5.02	912	0	0	0	462	495	Carbamidomethylation	PEAKS DB
K.LKPGAEC(+57.02)GNGLC(+57.02)C(+57.02)YQC(+57.02)K.I	N	50.91	2013.8529	17	-1.7	672.2905	3	5.69	1127	0	0	0	435	451	Carbamidomethylation	PEAKS DB
K.LKPGAEC(+57.02)GN(+.98)GLC(+57.02)C(+57.02)YQC(+57.02)K.I	N	49.78	2014.8369	17	0.2	672.6197	3	5.85	1177	0	0	0	435	451	Carbamidomethylation; Deamidation (NQ)	PEAKS PTM
R.ANVAKDS(-18.01)C(+57.02)FQENLKGSYYGYC(+57.02)R.K	Y	49.40	2611.1584	22	-10.9	653.7898	4	7.47	1692	0	0	0	518	539	Dehydration; Carbamidomethylation	PEAKS PTM
R.ANVAKDSC(+57.02)FQENLKGSYYGYC(+57.02)RK.E	Y	48.03	2757.2639	23	-15.8	552.4514	5	6.94	1523	0	0	0	518	540	Carbamidomethylation	PEAKS DB
K.DSC(+57.02)FQENLKGSYYGYC(+57.02)RK.E	N	46.62	2273.9834	18	0.9	759.0024	3	7.31	1643	0	0	0	523	540	Carbamidomethylation	PEAKS DB
K.DSC(+57.02)FQENLK.G	N	46.22	1139.4917	9	-3.1	570.7513	2	6.05	1245	0	0	0	523	531	Carbamidomethylation	PEAKS DB
R.LFC(+57.02)LNNSPR.N	N	46.06	1119.5494	9	3.8	560.7841	2	8.34	1952	0	0	0	558	566	Carbamidomethylation	PEAKS DB

Peptide	Uniq	-10lgP	Mass	Length	ppm	m/z	z	RT	Scan	Area SVMP	#Feature	#Feature SVMP	Start	End	PTM	Found By
R.NQC(+57.02)ISLFGSRANVAKDSC(+57.02)FQENLK.G	Y	44.27	2785.3276	24	-3.3	697.3369	4	9.14	2193	0	0	0	508	531	Carbamidomethylation	PEAKS DB
R.NQC(+57.02)ISLFGSRANVAKDSC(+57.02)FQENLKGSYYGYC(+57.02)R.K	Y	43.75	3791.7241	32	7.6	948.9456	4	9.52	2313	0	0	0	508	539	Carbamidomethylation	PEAKS DB
R.N(+27.99)QC(+57.02)ISLFGSR.A	N	43.73	1208.5608	10	-2.9	605.2859	2	9.41	2277	0	0	0	508	517	Formylation; Carbamidomethylation	PEAKS PTM
K.SHDNALLFTDMR.F	N	43.66	1418.6613	12	-5.9	710.3337	2	9.14	2194	0	0	0	282	293		PEAKS DB
K.SHDNALLFTDM(+15.99)R.F	N	43.35	1434.6561	12	-4.4	718.3322	2	7.56	1723	0	0	0	282	293	Oxidation (M)	PEAKS PTM
R.SVEIVQEQGNR.N	Y	43.28	1257.6313	11	-2.6	629.8213	2	5.76	1148	0	0	0	313	323		PEAKS DB
R.SVEIVQEQGNRNFK.T	Y	41.84	1646.8376	14	-3.1	824.4235	2	6.08	1253	0	0	0	313	326		PEAKS DB
R.ANVAKD(-18.01)SC(+57.02)FQENLKGSYYGYC(+57.02)R.K	Y	40.85	2611.1584	22	-10.5	653.7900	4	7.49	1698	0	0	0	518	539	Dehydration; Carbamidomethylation	PEAKS PTM
R.NQC(+57.02)ISLFGSR.A	N	40.29	1180.5659	10	-5.1	591.2872	2	8.43	1977	0	0	0	508	517	Carbamidomethylation	PEAKS DB
R.LFC(+57.02)LN(+.98)NSPR.N	N	40.10	1120.5336	9	5.3	561.2770	2	8.31	1944	0	0	0	558	566	Carbamidomethylation; Deamidation (NQ)	PEAKS PTM
R.L(+27.99)FC(+57.02)LNNSPR.N	N	39.12	1147.5444	9	-5.6	574.7763	2	10.56	2623	0	0	0	558	566	Formylation; Carbamidomethylation	PEAKS PTM

Peptide	Uniq	-10lgP	Mass	Length	ppm	m/z	z	RT	Scan	Area SVMP	#Feature	#Feature SVMP	Start	End	PTM	Found By
N.VAKDSC(+57.02)FQENLKGSYYGYC(+57.02)R.K	N	38.29	2444.0889	20	-28.5	815.6804	3	7.88	1817	0	0	0	520	539	Carbamidomethylation	PEAKS DB
R.ARDEC(+57.02)DVPEHC(+57.02)TGQSAEC(+57.02)PRDQLQQN(+.98)GKPC(+57.02)QNNR.G	Y	37.14	4054.7122	34	5.6	811.9542	5	5.14	957	0	0	0	462	495	Carbamidomethylation; Deamidation (NQ)	PEAKS PTM
R.KIPC(+57.02)APQDVKC(+57.02)GR.L	N	36.91	1527.7650	13	2.1	510.2634	3	4.86	862	0	0	0	545	557	Carbamidomethylation	PEAKS DB
R.KIPC(+57.02)APQDVK.C	N	36.61	1154.6117	10	0.8	578.3136	2	5.07	933	0	0	0	545	554	Carbamidomethylation	PEAKS DB
R.GYC(+57.02)YNGDC(+57.02)PIMR.N	N	36.46	1504.5897	12	-5.9	753.2977	2	7.04	1553	0	0	0	496	507	Carbamidomethylation	PEAKS DB
S.Q(-17.03)LVSTSAQFNK.I	N	36.44	1204.6088	11	-2.3	603.3103	2	7.90	1823	0	0	0	188	198	Pyro-glu from Q	PEAKS PTM
R.KSHDNALLFTDMR.F	N	35.58	1546.7562	13	3.3	516.5944	3	8.33	1947	0	0	0	281	293		PEAKS DB
K.LKPGAEC(+57.02)GNGLC(+57.02)C(+57.02)YQC(+57.02)KIK.T	N	35.58	2255.0320	19	-2.3	564.7640	4	6.22	1302	0	0	0	435	453	Carbamidomethylation	PEAKS DB
K.SHDNALLFTDMR(+43.04).F	N	34.81	1461.7034	12	-31.4	488.2264	3	7.78	1790	0	0	0	282	293	Carboxyl modification with ethanolamine	PEAKS PTM
R.KSHDNALLFTDMR(+43.04).F	N	34.30	1589.7983	13	-26.7	795.8853	2	7.11	1579	0	0	0	281	293	Carboxyl modification with ethanolamine	PEAKS PTM
R.KSHDNALLFTDM(+15.99)R.F	N	33.62	1562.7511	13	3.2	521.9260	3	6.85	1495	0	0	0	281	293	Oxidation (M)	PEAKS PTM
R.S(+27.99)VEIVQEQGNRNFK.T	Y	33.62	1674.8325	14	-7.9	838.4169	2	7.60	1734	0	0	0	313	326	Formylation	PEAKS PTM

Peptide	Uniq	-10lgP	Mass	Length	ppm	m/z	z	RT	Scan	Area SVMP	#Feature	#Feature SVMP	Start	End	PTM	Found By
K.C(-18.01)GRLFC(+57.02)LNNSPR.N	N	32.74	1417.6707	12	10.4	709.8500	2	7.59	1732	0	0	0	555	566	Dehydration (C@N-term); Carbamidomethylation	PEAKS PTM
R.N(+27.99)QC(+57.02)ISLFGSRANVAK.D	Y	30.39	1691.8413	15	-0.1	846.9279	2	9.08	2172	0	0	0	508	522	Formylation; Carbamidomethylation	PEAKS PTM
K.IPC(+57.02)APQDVKC(+57.02)GR.L	N	30.22	1399.6700	12	3.9	467.5658	3	5.15	958	0	0	0	546	557	Carbamidomethylation	PEAKS DB
R.RARDEC(+57.02)DVPEHC(+57.02)TGQSAEC(+57.02)PRDQLQQNGKPC(+57.02)QNNR.G	Y	29.59	4209.8291	35	5.7	842.9779	5	4.83	853	0	0	0	461	495	Carbamidomethylation	PEAKS DB
K.LKPGAE(+57.02)CGNGLC(+57.02)C(+57.02)YQC(+57.02)K.I	N	29.24	2013.8529	17	2.4	672.2932	3	5.68	1122	0	0	0	435	451	Carbamidomethylation (DHKE, X@N-term); Carbamidomethylation	PEAKS PTM
R.GYC(+57.02)YN(+.98)GDC(+57.02)PIMR.N	N	28.89	1505.5737	12	-3.3	753.7917	2	7.02	1550	0	0	0	496	507	Carbamidomethylation; Deamidation (NQ)	PEAKS PTM
R.ANVAKDS(-18.01)C(+57.02)FQENLK.G	Y	28.26	1604.7616	14	7.3	535.9317	3	5.95	1212	0	0	0	518	531	Dehydration; Carbamidomethylation	PEAKS PTM
R.NQC(+57.02)ISLFGSRAN(+.98)VAKDSC(+57.02)FQENLKGSYYGYC(+57.02)R.K	Y	28.19	3792.7083	32	10.4	759.5568	5	9.50	2304	0	0	0	508	539	Carbamidomethylation;	PEAKS PTM

Peptide	Uniq	-10lgP	Mass	Length	ppm	m/z	z	RT	Scan	Area SVMP	#Feature	#Feature SVMP	Start	End	PTM	Found By
															Deamidation (NQ)	
R.GYC(+57.02)YN(+.98)GDC(+57.02)PIMRNQC(+57.02)ISLFGSR.A	N	27.91	2668.1292	22	1.7	890.3851	3	10.45	2592	0	0	0	496	517	Carbamidomethylation; Deamidation (NQ)	PEAKS PTM
R.GYC(+57.02)YNGDC(+57.02)PIM(-48.00)R.N	N	27.63	1456.5864	12	3.5	486.5378	3	5.54	1075	0	0	0	496	507	Carbamidomethylation; Dethiomethyl	PEAKS PTM
R.ARDEC(+57.02)DVPEHC(+57.02)TGQSAEC(+57.02)PR.D	Y	27.62	2372.9531	20	-7.2	594.2413	4	4.20	738	0	0	0	462	481	Carbamidomethylation	PEAKS DB
K.DSC(+57.02)FQENLKGSYYGYC(+57.02)RKEN(+.98)GR.K	N	27.48	2731.1755	22	-1.3	683.8003	4	7.11	1578	0	0	0	523	544	Carbamidomethylation; Deamidation (NQ)	PEAKS PTM
R.GYC(+57.02)YNGDC(+57.02)PIMRNQC(+57.02)ISLFGSR.A	N	27.35	2667.1450	22	0.2	890.0558	3	10.33	2559	0	0	0	496	517	Carbamidomethylation	PEAKS DB
K.LKPGAEC(+57.02)GN(-17.03)GLC(+57.02)C(+57.02)YQC(+57.02)K.I	N	27.15	1996.8263	17	3.7	666.6185	3	6.12	1268	0	0	0	435	451	Carbamidomethylation; Ammonia-loss (N)	PEAKS PTM
R.LFC(+57.02)LN(-17.03)NSPR.N	N	26.31	1102.5229	9	-6.0	552.2654	2	7.65	1749	0	0	0	558	566	Carbamidomethylation; Ammonia-loss (N)	PEAKS PTM
R.ANVAK(+298.19)DSC(+57.02)FQENLKGSYYGYC(+57.02)R.K	Y	25.63	2927.3621	22	-27.7	732.8275	4	7.52	1710	0	0	0	518	539	Levuglandinyl-lysine anhyropyrrole adduct; Carbamid	PEAKS PTM

Peptide	Uniq	-10lgP	Mass	Length	ppm	m/z	z	RT	Scan	Area SVMP	#Feature	#Feature SVMP	Start	End	PTM	Found By
															omethylation	
R.GYC(+57.02)YNGDC(+57.02)PIM(+15.99)R.N	N	25.20	1520.5846	12	1.7	761.3008	2	6.08	1255	0	0	0	496	507	Carbamidomethylation; Oxidation (M)	PEAKS PTM
R.NQC(+57.02)ISLFGSRANVAK.D	Y	25.15	1663.8464	15	6.4	832.9359	2	8.03	1863	0	0	0	508	522	Carbamidomethylation	PEAKS DB
**total 52 peptides**																

ii. Matched peptides for RvMP light chain subunit (Q7LZ61).

Peptide	Uniq	-10lgP	Mass	Length	ppm	m/z	z	RT	Fraction	Scan	Area SVMP	#Feature	#Feature SVMP	Start	End	PTM	Found By
A.VLDC(+57.02)PSGWLSYEQHC(+57.02)YK.G	Y	61.43	2140.9346	17	-4.2	714.6492	3	9.55	5	2322	0	0	0	24	40	Carbamidomethylation	PEAKS DB
K.ALAEESYC(+57.02)LIMITHEKEWK.S	Y	56.33	2350.1338	19	1.6	784.3865	3	10.63	5	2642	0	0	0	114	132	Carbamidomethylation	PEAKS DB
K.S(+43.01)MTC(+57.02)NFIAPVVC(+57.02)K.F	Y	48.40	1568.7150	13	0.9	785.3655	2	8.79	5	2087	0	0	0	133	145	Carbamylation; Carbamidomethylation	PEAKS PTM
K.S(+71.04)MTC(+57.02)NFIAPVVC(+57.02)KF	Y	46.97	1743.8147	14	-22.4	872.8951	2	12.12	5	3027	0	0	0	133	146	Propionamide (K, X@N-term); Carbamidomethylation	PEAKS PTM
K.YKALAEESYC(+57.02)LIMITHEKEWK.S	Y	46.53	2641.2920	21	-0.9	661.3297	4	10.56	5	2622	0	0	0	112	132	Carbamidomethylation	PEAKS DB
K.S(+43.01)MTC(+57.02)NFIAPVVC(+57.02)KF	Y	45.04	1715.7833	14	4.1	572.9374	3	11.15	5	2789	0	0	0	133	146	Carbamylation; Carbamidomethylation	PEAKS PTM
K.SMTC(+57.02)NFIAPVVC(+57.02)K.F	Y	41.93	1525.7091	13	-2.1	763.8602	2	9.45	5	2288	0	0	0	133	145	Carbamidomethylation	PEAKS DB
K.SMTC(+57.02)NFIAPVVC(+57.02)KF	Y	41.55	1672.7776	14	-3.8	837.3929	2	11.67	5	2919	0	0	0	133	146	Carbamidomethylation	PEAKS DB
K.SM(+15.99)TC(+57.02)NFIAPVVC(+57.02)K.F	Y	41.20	1541.7041	13	-0.4	771.8590	2	8.70	5	2060	0	0	0	133	145	Oxidation (M); Carbamidomethylation	PEAKS PTM
K.SM(-48.00)TC(+57.02)NFIAPVVC(+57.02)K.F	Y	36.35	1477.7058	13	-0.2	493.5758	3	7.72	5	1772	0	0	0	133	145	Dethiomethyl; Carbamidomethylation	PEAKS PTM
K.SM(+15.99)TC(+57.02)NFIAPVVC(+57.02)KF	Y	33.60	1688.7725	14	2.2	845.3953	2	11.07	5	2762	0	0	0	133	146	Oxidation (M); Carbamidomethylation	PEAKS PTM
K.ALAEE(+57.02)SYC(+57.02)LIMITHEKEWK.S	Y	31.48	2407.1553	19	0.1	602.7961	4	9.23	5	2222	0	0	0	114	132	Carbamidomethylation (DHKE, X@N-term); Carbamidomethylation	PEAKS PTM

Peptide	Uniq	-10lgP	Mass	Length	ppm	m/z	z	RT	Fraction	Scan	Area SVMP	#Feature	#Feature SVMP	Start	End	PTM	Found By
K.ALAEESYC(+57.02)LIMITHEK(+43.01).E	Y	30.05	1949.9226	16	-0.9	650.9809	3	9.92	5	2444	0	0	0	114	129	Carbamidomethylation; Carbamylation	PEAKS PTM
K.S(+43.01)MTC(+57.02)NFIAPVVC(+57.02)K(+57.02)F	Y	26.79	1772.8048	14	-2.2	887.4077	2	11.13	5	2780	0	0	0	133	146	Carbamylation; Carbamidomethylation; Carbamidomethylation (DHKE, X@N-term)	PEAKS PTM
K.SM(-48.00)TC(+57.02)NFIAPVVC(+57.02)KF	Y	26.43	1624.7742	14	-2.8	813.3921	2	10.09	5	2493	0	0	0	133	146	Dethiomethyl; Carbamidomethylation	PEAKS PTM
N.F(+57.02)IAPVVC(+57.02)KF	Y	26.00	1136.6052	9	-4.3	569.3074	2	10.77	5	2678	0	0	0	138	146	Carbamidomethylation (DHKE, X@N-term); Carbamidomethylation	PEAKS PTM
K.S(+100.02)MTC(+57.02)NFIAPVVC(+57.02)K.F	Y	25.88	1625.7252	13	7.5	813.8760	2	8.90	5	2119	0	0	0	133	145	Methylmalonylation on Serine; Carbamidomethylation	PEAKS PTM
A.VLDC(+57.02)PSGWLSYEQH.C	Y	25.78	1689.7457	14	-0.2	845.8800	2	9.79	5	2397	0	0	0	24	37	Carbamidomethylation	PEAKS DB
K.S(+57.02)MTC(+57.02)NFIAPVVC(+57.02)KF	Y	25.76	1729.7990	14	1.6	865.9081	2	11.83	5	2960	0	0	0	133	146	Carbamidomethylation (DHKE, X@N-term); Carbamidomethylation	PEAKS PTM
M.T(+57.02)C(+57.02)NFIAPVVC(+57.02)KF	Y	25.72	1511.7264	12	-3.2	756.8680	2	11.19	5	2800	0	0	0	135	146	Carbamidomethylation (DHKE, X@N-term); Carbamidomethylation	PEAKS PTM
K.S(+42.01)M(+15.99)TC(+57.02)NFIAPVVC(+57.02)K.F	Y	25.70	1583.7146	13	-4.6	792.8610	2	8.38	5	1962	0	0	0	133	145	Acetylation (N-term); Oxidation (M); Carbamidomethylation	PEAKS PTM
**total 21 peptides**																	

Supplementary Data S2: Sequence of $RvPLA_2$

a. Protein identification for RvPLA2. P31100 was determined to be the identity of PLA_2. The determination was made based on analysis of -10lgP value, number of unique peptides and matched origin species.

Protein Group	Protein ID	Accession	-10lgP	Coverage (%)	Coverage (%) P1	Area P1	#Peptides	#Unique	#Spec P1	PTM	Avg. Mass	Description
**1**	5933	P31100|PA2A7_DABSI	220.06	88	88	0	17	13	80	Y	15421	Acidic phospholipase A2 RV-7 OS=Daboia siamensis PE=1 SV=1
**1**	5932	tr|B3RFI6|B3RFI6_9SAUR	220.06	88	88	0	17	13	80	Y	15421	Acidic phospholipase A2 OS=Daboia russellii limitis PE=2 SV=1
**2**	5929	tr|B2YHV1|B2YHV1_DABSI	199.54	75	75	0	18	18	77	Y	15555	Basic phospholipase A2 OS=Daboia siamensis GN=PLA2S1 PE=2 SV=1
**2**	5930	Q02471|PA2B4_DABSI	199.54	75	75	0	18	18	77	Y	15555	Basic phospholipase A2 RV-4 OS=Daboia siamensis PE=1 SV=1
**3**	5935	tr|Q6A3N6|Q6A3N6_VIPAZ	121.89	50	50	0	6	2	13	Y	15421	Vaspin acidic subunit (2) isoform OS=Vipera aspis zinnikeri PE=2 SV=1

Supporting Peptides:

Peptide	Uniq	-10lgP	Mass	Length	ppm	m/z	z	RT	Fraction	Scan	Source File	Area P1	#Feature	Start	End	PTM	Found By
K.EVVHSYAIYGC(+57.02)YC(+57.02)GWGGQGR.A	Y	72.37	2317.9998	20	5.2	773.6779	3	8.77	3	1977	P1-aMSMS-30(47)-1uL.d	0	0	32	51	Carbamidomethylation	PEAKS DB
K.TGKEVVHSYAIYGC(+57.02)YC(+57.02)GWGGQGR.A	Y	70.21	2604.1638	23	0.3	869.0621	3	8.30	3	1847	P1-aMSMS-30(47)-1uL.d	0	0	29	51	Carbamidomethylation	PEAKS DB
K.TATYSYSFEN(+.98)GDIVC(+57.02)GDNDLC(+57.02)LR.T	Y	52.73	2670.1213	23	0.0	891.0477	3	10.32	3	2457	P1-aMSMS-30(47)-1uL.d	0	0	77	99	Deamidation (NQ); Carbamidomethylation	PEAKS PTM
G.NLFQFGEMILEKTGK.E	Y	52.50	1753.9072	15	2.0	877.9626	2	13.34	3	3183	P1-aMSMS-30(47)-1uL.d	0	0	17	31		PEAKS DB
R.TVC(+57.02)EC(+57.02)DRAAAIC(+57.02)LGQNVNTYDKNYEYYSISHC(+57.02)TEESEQC(+57.02)	Y	47.30	4736.9458	39	-3.7	1185.2394	4	9.21	3	2117	P1-aMSMS-30(47)-1uL.d	0	0	100	138	Carbamidomethylation	PEAKS DB
D.ATDRC(+57.02)C(+57.02)FVHDC(+57.02)C(+57.02)YGTVNDC(+57.02)NPK.T	Y	45.62	2748.0608	22	3.6	688.0250	4	6.00	3	1172	P1-aMSMS-30(47)-1uL.d	0	0	55	76	Carbamidomethylation	PEAKS DB
R.C(+57.02)C(+57.02)FVHDC(+57.02)C(+57.02)YGTVNDC(+57.02)NPK.T	Y	45.06	2304.8479	18	-0.4	769.2896	3	6.19	3	1237	P1-aMSMS-30(47)-1uL.d	0	0	59	76	Carbamidomethylation	PEAKS DB
R.AAAIC(+57.02)LGQNVNTYDKNYEYYSISHC(+57.02)TEESEQC(+57.02)	Y	41.95	3816.5977	32	-1.3	1273.2048	3	9.73	3	2283	P1-aMSMS-30(47)-1uL.d	0	0	107	138	Carbamidomethylation	PEAKS DB

Peptide	Uniq	-10lgP	Mass	Length	ppm	m/z	z	RT	Fraction	Scan	Source File	Area P1	#Feature	Start	End	PTM	Found By
G.NLFQFGEMILEK.T	Y	41.68	1467.7432	12	7.1	734.8841	2	13.59	3	3242	P1-aMSMS-30(47)-1uL.d	0	0	17	28		PEAKS DB
Y.AIYGC(+57.02)YC(+57.02)GWGGQGR.A	N	41.01	1603.6660	14	3.0	802.8427	2	7.75	3	1692	P1-aMSMS-30(47)-1uL.d	0	0	38	51	Carbamidomethylation	PEAKS DB
K.NYEYYSISHC(+57.02)TEESEQC(+57.02)	N	40.36	2197.8206	17	-2.2	1099.9152	2	6.89	3	1442	P1-aMSMS-30(47)-1uL.d	0	0	122	138	Carbamidomethylation	PEAKS DB
K.TATYSYSFEN(+.98)GDIVC(+57.02)GD(-18.01)NDLC(+57.02)LR.T	Y	36.71	2652.1108	23	1.1	885.0452	3	10.53	3	2522	P1-aMSMS-30(47)-1uL.d	0	0	77	99	Deamidation (NQ); Carbamidomethylation; Dehydration	PEAKS PTM
K.EVVH(+57.02)SYAIYGC(+57.02)YC(+57.02)GWGGQGR.A	Y	32.74	2375.0212	20	-1.1	792.6801	3	8.76	3	1973	P1-aMSMS-30(47)-1uL.d	0	0	32	51	Carbamidomethylation (DHKE, X@N-term); Carbamidomethylation	PEAKS PTM
R.TVC(+57.02)EC(+57.02)DRAAAIC(+57.02)LGQNVNTYDK.N	Y	31.80	2557.1360	22	-15.5	853.3727	3	7.82	3	1714	P1-aMSMS-30(47)-1uL.d	0	0	100	121	Carbamidomethylation	PEAKS DB
K.EVVHSYAIYGC(+57.02)Y(-2.02)C(+57.02)GWGGQGR.A	Y	31.56	2315.9841	20	-6.2	772.9972	3	8.82	3	1994	P1-aMSMS-30(47)-1uL.d	0	0	32	51	Carbamidomethylation; 2-	PEAKS PTM

Peptide	Uniq	-10lgP	Mass	Length	ppm	m/z	z	RT	Fraction	Scan	Source File	Area P1	#Feature	Start	End	PTM	Found By
																amino-3-oxo-butanoic_acid	
R.AQD(+57.02)ATDRC(+57.02)C(+57.02)FVHDC(+57.02)C(+57.02)YGTVNDC(+57.02)NPK.T	Y	31.13	3119.2048	25	6.2	780.8133	4	6.13	3	1212	P1-aMSMS-30(47)-1uL.d	0	0	52	76	Carbamidomethylation (DHKE, X@N-term); Carbamidomethylation	PEAKS PTM
Y.SFEN(+.98)GDIVC(+57.02)GDNDLC(+57.02)LR.T	N	30.37	1983.8302	17	4.7	992.9271	2	9.29	3	2148	P1-aMSMS-30(47)-1uL.d	0	0	83	99	Deamidation (NQ); Carbamidomethylation	PEAKS PTM
R.C(+57.02)C(+57.02)FVH(+40.03)DC(+57.02)C(+57.02)YGTVNDC(+57.02)NPK.T	Y	30.30	2344.8792	18	-11.5	782.6246	3	7.71	3	1679	P1-aMSMS-30(47)-1uL.d	0	0	59	76	Carbamidomethylation; Propionaldehyde +40	PEAKS PTM
R.AQDATDRC(+57.02)C(+57.02)FVH(+57.02)DC(+57.02)C(+57.02)YGTVNDC(+57.02)NPK.T	Y	29.73	3119.2048	25	8.0	780.8147	4	6.11	3	1209	P1-aMSMS-30(47)-1uL.d	0	0	52	76	Carbamidomethylation; Carbamidomethylation (DHKE, X@N-term)	PEAKS PTM
Y.SFED(sub N)GDIVC(+57.02)GDNDLC(+57.02)LR.T	N	29.68	1983.8302	17	5.5	992.9279	2	9.27	3	2143	P1-aMSMS-	0	0	83	99	Carbamidomethylation	SPIDER

Peptide	Uniq	-10lgP	Mass	Length	ppm	m/z	z	RT	Fraction	Scan	Source File	Area P1	#Feature	Start	End	PTM	Found By
											30(47)-1uL.d						
R.AQDATDRC(+57.02)C(+57.02)FVHDC(+57.02)C(+57.02)YGT(-18.01)VNDC(+57.02)NPK.T	Y	27.36	3044.1729	25	9.9	762.0580	4	6.17	3	1230	P1-aMSMS-30(47)-1uL.d	0	0	52	76	Carbamidomethylation; Dehydration	PEAKS PTM
R.AAAIC(+57.02)LGQNVNTYDK.N	Y	26.88	1636.7878	15	3.0	819.4037	2	7.52	3	1627	P1-aMSMS-30(47)-1uL.d	0	0	107	121	Carbamidomethylation	PEAKS DB
R.C(+57.02)(+57.02)C(+57.02)FVHDC(+57.02)C(+57.02)YGTVNDC(+57.02)NPK.T	Y	25.46	2361.8694	18	-2.6	788.2950	3	6.53	3	1344	P1-aMSMS-30(47)-1uL.d	0	0	59	76	Carbamidomethylation; Carbamidomethylation (DHKE, X@N-term)	PEAKS PTM
D.RC(+57.02)C(+57.02)FVHDC(+57.02)C(+57.02)YGTVNDC(+57.02)NPK.T	Y	25.37	2460.9490	19	10.1	616.2507	4	5.72	3	1084	P1-aMSMS-30(47)-1uL.d	0	0	58	76	Carbamidomethylation	PEAKS DB
K.EVVHSY(+125.90)AIYGC(+57.02)YC(+57.02)GWGGQGR.A	Y	25.14	2443.8962	20	4.3	815.6428	3	9.94	3	2349	P1-aMSMS-30(47)-1uL.d	0	0	32	51	Iodination; Carbamidomethylation	PEAKS PTM
**total 27 peptides**																	
